# The Context of Application of Biosecurity for Control of African Swine Fever in Smallholder Pig Systems: *Current Gaps and Recommendations*

**DOI:** 10.3389/fvets.2021.689811

**Published:** 2021-08-02

**Authors:** Florence Mutua, Michel Dione

**Affiliations:** ^1^Animal and Human Health Program, International Livestock Research Institute, Nairobi, Kenya; ^2^Animal and Human Health Program, International Livestock Research Institute, Dakar, Senegal

**Keywords:** biosecurity, African swine fever, African swine fever virus, disease outbreaks, smallholder pig systems

## Abstract

African swine fever (ASF) is a highly fatal disease of pigs. It is a threat to the pig industry as it lowers production and significantly impacts on livelihoods. ASF has no cure and a vaccine against it is yet to be developed. Outbreaks continue to be reported in Africa and Asia, where the setting of the pig value chain (farm, market, and slaughter practices) coupled with the risky behaviors of actors, contribute to persistence of the virus in pig populations. The role of these factors in the epidemiology of the disease is reviewed with a focus on smallholder pig systems in Africa. Biosecurity at the farm level is particularly emphasized, and factors influencing its adoption highlighted. Socio-cultural factors and weaknesses at the disease control policy level are critical and should not be ignored. Gender and equity are important aspects and ought to be considered in discussions to improve the sector. The findings are expected to define priorities for interventions to improve pig productivity (as these regions wait for the vaccine to be developed).

## Introduction

The world population continues to increase, and the food insecurity challenge has been worsened by the COVID-19 pandemic. Livestock are important livelihood assets especially for the poor who use the income from their sales to meet important household needs. Animal source foods are nutrient dense. They are a source of protein and provide micronutrients in forms that are available for the body (iron, calcium, vitamin B12) (1). Demand for food has been growing in developing countries (2) and this trend is likely to continue in the future, given the predicted increases in human population, challenge of urbanization and rising incomes.

Small livestock species, such as pigs, can easily be raised by people with limited resources (3) providing opportunities for regular supply of protein. Also, the demand for pork has increased and many rural and peri-urban communities have discovered the cost-effectiveness of keeping pigs (4). Because of this, pigs in many developing countries are being reared as important income sources (3). Pork is one of the cheapest forms of animal proteins (5). It is reportedly the world‘s most widely eaten meat (accounting for over 36% of total meat eaten) (6). Consumption is increasing (7) and has been projected to increase by 154.9% in sub-saharian Africa between 2000 and 2030 (8).

The increase in demand for pork is driving growth of the sector, presenting opportunities for farmers to invest and gain from pig production. In sub-Saharan Africa (SSA), the majority of pigs are kept by smallholder farmers who manage them either under extensive or semi extensive systems (9, 10). Pigs are easily integrated into small-scale farming systems. They can be fed with by-products from crops that cannot be consumed or used more efficiently by households. Their manure can be used as fertilizer as well as for energy production (11). Pigs can farrow at least two times in a year and have the potential to yield large litter sizes. Offspring can be sold or reared to maturity. Apart from regions with cultural and religious reservations toward pork, pigs are a potentially viable and valuable investment option for producers, and an important diversification enterprise especially for women (12, 13). The full potential of pig production in SSA is yet to be tapped and this is mainly because of the challenges of feeding and disease.

African swine fever (ASF) is a threat to the pig industry, especially in countries that are still developing (14–17). The impact of ASF is felt more in countries with high pig numbers including Uganda which has the second highest pig population density in sub-Saharan Africa (16). The high mortality and ability to spread to non-infected areas makes ASF a concern to the pig industry, globally (18). Its widespread occurrence implies gaps in disease prevention and control. Several factors are thought to contribute to ASF outbreaks among them poor husbandry practices, weak implementation of biosecurity measures (including allowing pigs to free range), inappropriate behaviors of value chain actors, particularly the illegal live pig trades that happen during outbreak (referred as “panic sales”), the inappropriate practices of pork butchers, and the low financial capability of farmers that limits how much they can invest in disease control (19, 20). Asymptomatic carriers remain a concern (21, 22); and if their role in the persistence of the virus in pig populations is fully demonstrated, the situation will further complicate implementation of control measures.

Although research on vaccine development has been ongoing for some time, neither a vaccine nor a therapeutic product for ASF are currently available, a situation that makes disease control more demanding. The impact of ASF can therefore only be minimized through the adoption of biosecurity measures (23, 24). It has been predicted that biosecurity measures implemented within 14 days of the onset of an epidemic can avert up to 74% of deaths due to ASF (25). Biosecurity measures are not adequately implemented in most smallholder pig farms and this is mainly because the farmers lack the capacity required to do so (26). Further, Nantima et al. (20) note that smallholders are unable to comply with biosecurity measures given the nature of their production system and mentions that adoption of biosecurity is only feasible for pigs that are confined (either housed or tethered) as opposed to those allowed to roam freely. While medium and large-scale farmers may have the capacity to invest in biosecurity measures, this is often not the case especially for small-scale farmers who prefer to keep few pigs at a time and may not confine them. The objective of our paper is to document and discuss the feasibility of biosecurity measures in smallholder pig systems in low income countries and provide recommendations how ASF can sustainably be controlled for the time being.

## Methodology for the Review

**Figure 1:**
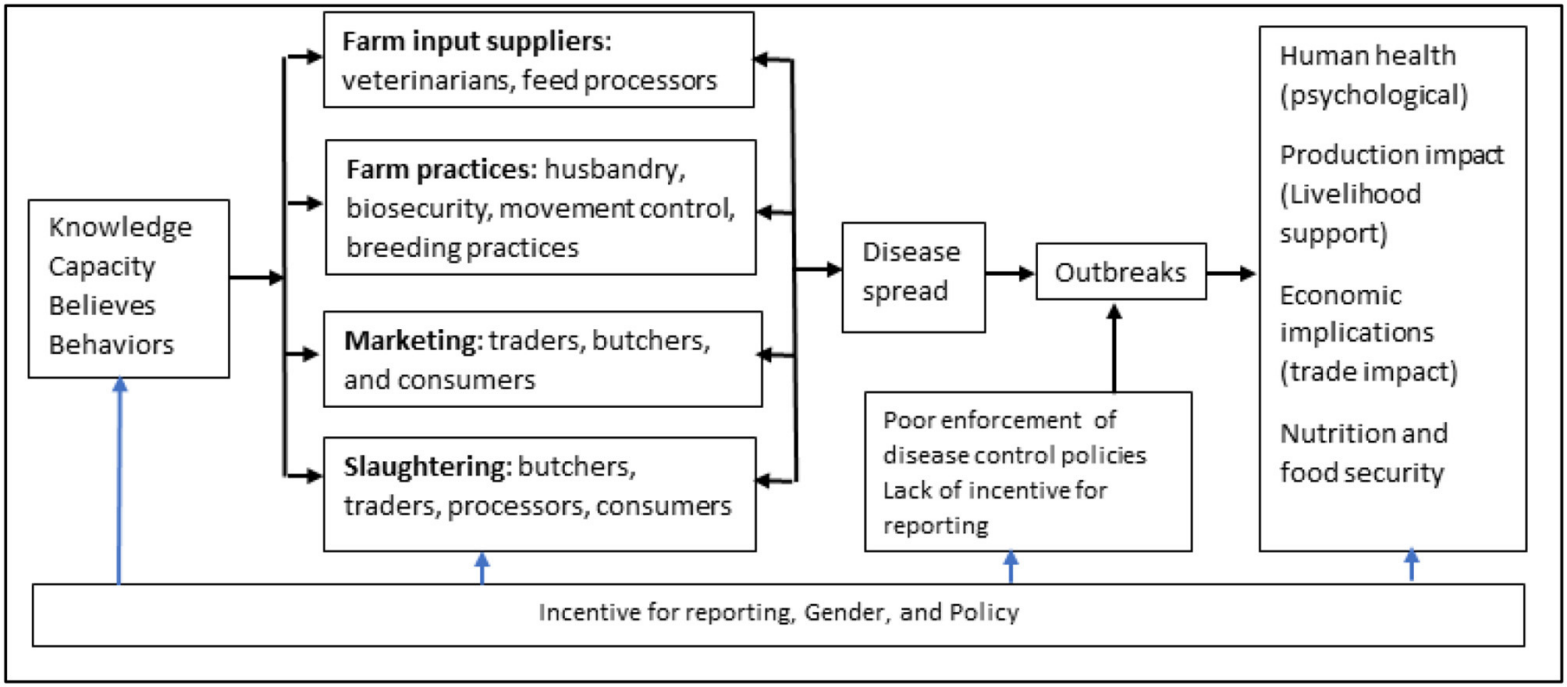
Framework used in the desk-based study.

The authors present a desk-based study. At the start of the study, a framework highlighting the factors associated with ASF virus spread was developed (Figure 1). It included key factors such as input supplies, farm level practices, marketing, processing, policy, as well as the impacts that ASF can have, especially in developing countries (trade, food security, and livelihoods). Gender was specifically considered as a cross-cutting elements given the roles women play in pig management and marketing. The discussion is framed around these key areas with an emphasis on biosecurity and what can be considered as bottlenecks in its implementation.

## Description of review findings

### Epidemiology of African Swine Fever

ASF is a contagious and highly lethal hemorrhagic disease of pigs and is considered the greatest obstacle to development of the industry in SSA (18, 27). ASF was first reported in East Africa but then spread to many other countries (28, 30), including Europe, South East Asia and the Caribbean region (28). Smallholder pig production in the African region is well described in previous studies (29, 31–37).

The causative agent, the African swine fever virus (ASFV), is a large, enveloped, double-stranded DNA virus (38). Epizootic outbreaks can arise in a number of ways. ASFV can be transmitted through direct contact with infected pigs (by the oral-nasal route or through skin abrasions) (39). The virus spreads effectively by contact via aerosol droplets and blood, feces and other virus-infected tissues (18). Wild suids in Africa can be persistently infected and develop few if any clinical signs and little or no viremia (39). In Europe, the wild boar suffers an acute disease similar to the domestic pig (38). Young warthogs develop a transient viremia that is sufficient to infect *Ornithodoros* moubata ticks when they feed on them. The *Ornithodoros* tick vector is thought to play an important role in virus transmission between warthog hosts (39). The sylvatic cycle involves warthogs and soft ticks. In Africa, transmission from warthogs, *Ornithodoros moubata* ticks or bush pigs, to domestic pigs is relatively infrequent and limited to village farms especially those in areas close to the wildlife reservoirs (40).

The incubation period in domestic pigs ranges from 5 to 15 days, and in clinical cases, there is fever (41–42°C for about 4 days), diarrhea, inappetence, incoordination, prostration, coma, and death (27). Vomiting, nasal and conjunctival discharge, dyspnea, anal and nasal hemorrhages can also be observed in some animals. Abortion is common in affected sows. In regions where ASF is absent, mortality rates often reach 100% (27), making it a highly dreaded disease. ASF cases can also be predominantly subclinical especially in endemic areas (41). Although early detection and laboratory diagnosis are essential for the control and management of the disease, resemblance with other hemorrhagic diseases of pigs including porcine reproductive and respiratory disease, erysipelas, and salmonellosis need to be considered (42, 43).

It has been noted that previous efforts to develop an ASF vaccine have not been successful (44). Vaccine development has been hindered by the limited knowledge regarding the disease and the virus strain variation (45). There is also no treatment for the disease. Suggested control measures include investing in quarantine facilities, banning free range pig systems, implementation of enhanced biosecurity, a ban on illegal import of pork and pork products, and the introduction of an improved surveillance system. In Europe, the main preventive measures are the use of animal identification and tracing systems, the enforcement of swill feeding bans, and containment of pigs to ensure they do not come into contact with pigs from other farms, feral pigs, wild boar, or their products (46). Livestock identification and traceability systems are virtually non-existent in many smallholder pig systems in Africa (47) and cannot be relied upon to reduce disease spread. Swill feeding can introduce disease in healthy populations (48). It is impossible to monitor its use at the household level although farmers can be requested to boil the swill before feeding it to their pigs, for about 30 min (49). Asking smallholders to confine their pigs will face some resistance as this imposes feeding obligations which they may not be ready to undertake (48). As such, a national policy which includes identifying sources of feedstuffs that are readily available and affordable, should be put in place (49). Stamping out is another approach but this also is not feasible especially in settings where ASF is endemic (as is the case in SSA). It involves (1) early detection, (2) enabling legislation for declaring national emergencies, (3) zoning of the country into infected zones, surveillance zones and free zones, (4) inspection and quarantine, (5) immediate slaughter of infected animals, (5) epidemiological surveillance, (6) safe burial of carcasses, (7) cleaning and disinfection of carcasses and (8) keeping previously infected premises and villages free of pigs (49). It has been observed that eradication of the disease from SSA is not an option given the involvement of African wild suids and ticks of the *Ornithodoros moubata* complex, in the epidemiology of the disease (48). It has also been noted that, in Africa, the domestic pig cycle is driven by poverty (30) hence the need to consider the role of other factors when designing interventions to control diseases involving pigs.

### Biosecurity Control Measures

Biosecurity is key in ensuring disease free farms, regions, and countries. Its adoption will not only reduce the risk of disease introduction significantly, but will also reduce the magnitude of the financial losses that may occur following introduction of the disease in susceptible pig populations (50). Biosecurity has been defined as the management of the risk of pests and diseases entering, emerging, establishing, or spreading and causing harm to animals, plants, human health, the economy, the environment, or the community (51). In an agricultural context, “biosecurity” refers to practices that control the spread of disease both into and within the farm (52). As observed by Villarroel et al. (53) and Laanen et al. (54), a key component of farm-level biosecurity is biocontainment or internal biosecurity, which has been described as the series of management practices that prevent the spread of infectious agents between animal groups in a farm or the management practices designed to prevent the infectious agent from leaving the farm.

There are three main levels of biosecurity (55): (1) segregation, the creation and maintenance of barriers to limit entry of infected animals and contaminated materials to a non-infected site. Segregation measures include controlling the entry of pigs from outside farms, markets or villages, implementing quarantine for newly purchased animals, limiting the number of sources of replacement stocks, fencing farm areas and controlling access for people, as well as that of birds, bats, rodents, cats and dogs, maintaining adequate distances between farms, providing footwear and clothing to be worn only on the farm, and using an all-in-all-out management system (56); (2) cleaning of materials, including vehicles and equipment that enter or leave a site, aiming to remove all visible dirt. It is expected that the cleaning will remove most of the contaminants; and (3) disinfection which, when properly applied, will inactivate any pathogen present on materials that have already been cleaned. Cleaning and disinfection measures may involve the use of high-pressure and low-pressure washers, targeting buildings on the premises, but also vehicles, equipment, clothing and footwear. Cleaning and disinfection procedures are thought to be fundamental for pathogen inactivation, to prevent disease spread, and to facilitate repopulation after an outbreak (56). Indeed, cleaning represents one of the most important steps in the cleaning and disinfection process (57). It removes over 90% of microorganisms when properly performed and improves the disinfection efficacy (58). Other biosecurity concerns that Chenais et al. (59) highlight include slaughtering of pigs that showed signs of the disease and gaps in handling of the waste, high turnover of staff causing biosecurity routines to be lost and the handling of waste water. Compliance with biosecurity protocols in smallholder pig systems is challenging, possibly more so in a country like Uganda where ASF is endemic, even for farms that are fenced off and may confine their pigs (59).

### Impact of African Swine Fever in Smallholder Pig Systems

African swine fever is a highly fatal disease of pigs. It has significant impacts on food security, income, and development. Because of a lack of epidemiological data, the impact of ASF is not well-understood, especially in SSA. The negative impacts are more significant in smallholder settings where pigs are traditionally raised and biosecurity systems are weak (60). According to Chenais et al. (61) assessing the economic impact of ASF in such systems is complicated, as the pigs are mostly reared as what could be seen as passive investments rather than being an active working capital.

Costs associated with ASF outbreaks are dependent on the nature of the virus and the degree to which susceptible populations are exposed (62). Sick pigs, as well as those that have been exposed to the virus, are often culled to reduce spread (63) and mitigate financial loss. Also, because of the uncertainty created following outbreaks, producers may be reluctant to increase their pig numbers (64). The result is a reduction in the amount of pork on the market and subsequently, an increase in price. In China, it is reported that the retail prices in the country rose by 78% heavily impacting consumers (65). It also has been observed that consumers are also likely to substitute pork with relatively cheaper products (66) further destabilizing the market. ASF has been circulating in domestic pig populations in Tanzania (67). A study involving 1085 households reported a mortality rate of 84% (range 46–97) (68). The authors found the average number of pigs lost per household to be 4 and this translated to a loss of Tsh 160.632 million (equivalent of USD 92,583 at a conversion rate of 1 USD = TSH 1,735, estimate for 2014 when the study was undertaken).

It has been reported that, by the end of 2019, due to ASF-outbreaks, the national pig herd in China was reduced by half (65). A study involving several countries in Europe found new ASF-events in the period between 2010 and 2019 to have reduced pork exports by almost 15% in the year after the cases had occurred (69). The feed sector was also affected, given the reduced demand. China's total consumption of animal feeds is said to have dropped by 17% in 2019 (65). Given the high mortality in pigs, and as an indirect result of the disease, staff employed in pig enterprises risk losing their jobs when outbreaks occur (59). The situation may be exacerbated if farmers decide to withdraw from production, especially in settings where pig production contributes significantly to local livelihoods.

Another concern is that producers in countries where the disease is endemic may not report all outbreaks to authorities (70) and sick pigs may be traded to reduce any losses due to ASF thereby increasing the spread of the disease. This was observed in Uganda where households that reported ASF outbreaks were found to consume meat more times per month compared to those that did not report any outbreaks (61).

### Farm and Value Chain Management Practices That Influence Occurrence of African Swine Fever

#### Pig Husbandry Practices

**Figure 2:**
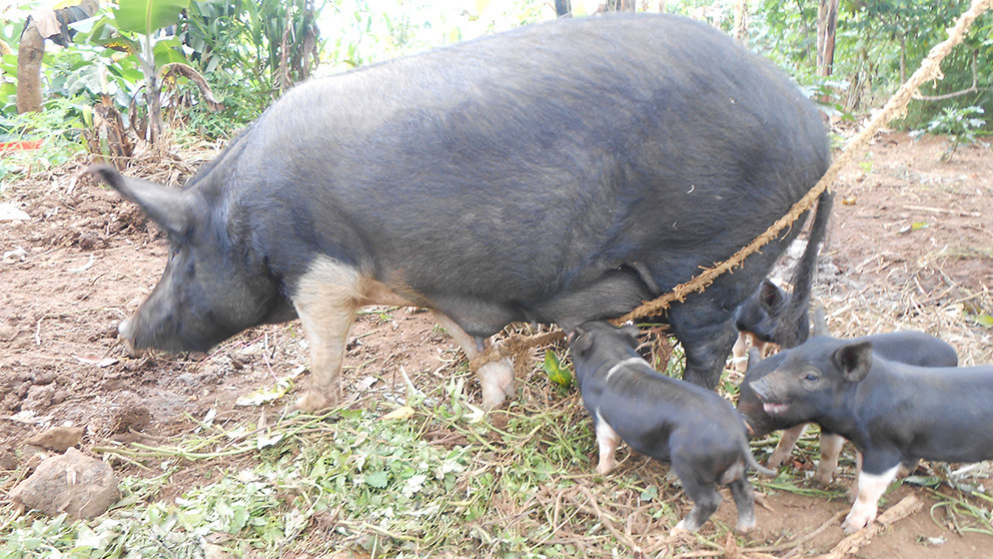
Tethered sow with piglets in Uganda (picture credit: Michel Dione, ILRI).

**Figure 3:**
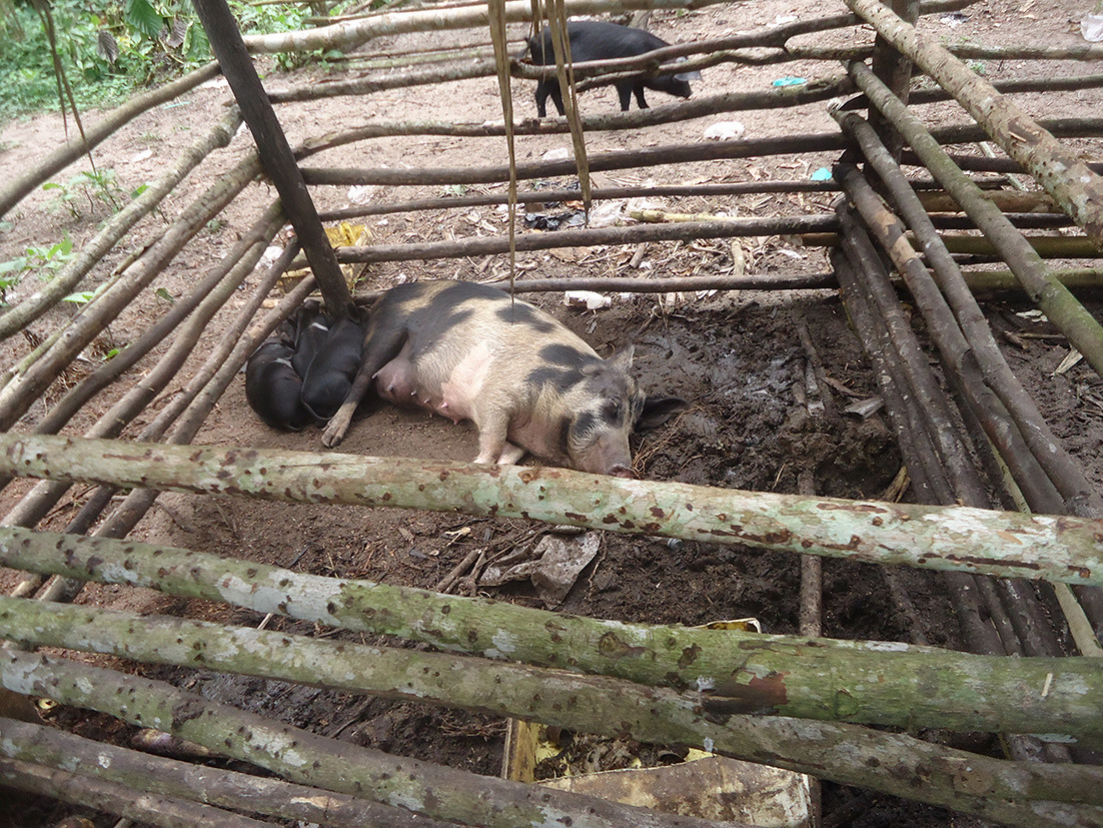
Housed sow with piglets in Uganda (picture credit: Michel Dione, ILRI).

**Figure 4:**
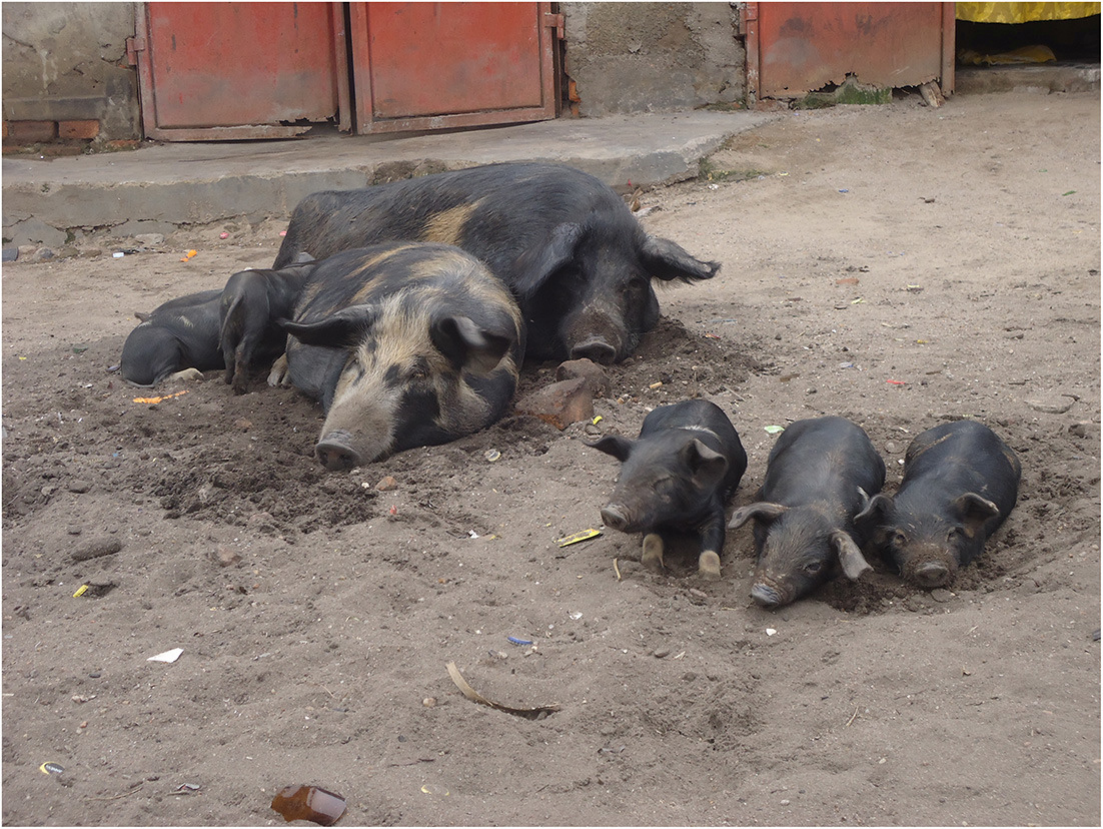
Free-ranging pig in Uganda (picture credit: Michel Dione, ILRI).

Pig production in many sub-Saharan countries is characterized by backyard farming of small number of animals, managed semi-intensively, seasonal confinement, free-roaming or tethering (Figures 2–4). As minimal health care is afforded to pigs in these systems, the burden of infections, especially in relation to helminthiasis (71) and respiratory diseases (36), is very high. Such systems are prone to ASF incursions with epidemic peaks observed throughout the year. In terms of confinement, it is the pigs in peri-urban and urban settings that are more likely to be confined (15, 33, 72–74). The confined pigs receive better care including medication as, and when required, and commercial feeds since the production is more market oriented.

#### Pig Feeding Systems

Field experiences show that pigs reared in extensive or semi-extensive systems are mostly fed on crop residues or forages, while those in peri-urban or urban areas have access to swill (i.e., the leftover food from owners and restaurants, which do not undergo any processing). Richer farmers may purchase commercially formulated feeds or raw materials such as maize, rice bran, etc. which they can use to formulate rations for their pigs. Feeding strategies change depending on availability of feed resources, which also follow a seasonal pattern (9, 34, 75). It is worth noting that ASF can be perpetuated among permanently confined pigs through swill feeding (76). The virus can survive in chilled meat or carcasses for up to 6 months, and at 4°C for two years. It has been shown to remain infective in smoked and salted pork. ASFV is highly resistant to putrefaction and can remain in feces for at least 11 days and in decomposed serum for 15 weeks (77). During an outbreak investigation in the central region in Uganda, ASFV was isolated from tissues of pigs that had died from ASF (78), suggesting that feeding pigs with contaminated materials or undercooked pork can predispose the animals to the disease. However, practices like processing swill by heating can kill the virus and consequently decrease the risk of virus transmission to healthy pigs. Niederwerder et al. (79) demonstrate that ASFV can easily be transmitted orally (although higher doses will be required for infection to occur through plant-based feed). In 2014, the introduction and spread of ASFV in Latvia was associated with failure to use simple biosecurity measures notably the feeding of virus-contaminated fresh grass or crops to naive pigs (80). It has also been demonstrated that ASFV can survive in feed ingredients (under simulated transboundary shipping models) (81) suggesting that ASFV spread might be attributed to less-recognized transmission routes such as feed or water (79). In smallholder systems, pig feeding strategies generally depend on availability of the feed resources and the ability of farmers to buy ingredients, which often are expensive. Financial constraints can lead to sub-optimal feeding practices, hence the risk for ASF can increase.

#### Movements of Pigs and Products

ASF has proved difficult to eradicate due to the movement of infected pigs and pig products (14, 82). Especially in East Africa, pig movements, due to trade and restocking, are the most common risk factors associated with the spread of ASF in small-holder systems (26, 83). In Uganda, movements of pigs and pork products were responsible for the vast majority of outbreaks (83). Animal loan practices for breeding purposes, such as sharing of boars and purchases can be a factor contributing to the transmission of ASF virus between farms, through direct pig- to-pig contact (84). Transboundary movements of pigs have also been associated with outbreaks at the borders of Uganda with 20.6% of reported outbreaks between 2001 and 2012 taking place in areas adjacent to international borders (14). Advanced genomic studies involving ASFV strains from Uganda, from outbreaks in 2007, identified 22 different tetrameric amino acid units, which were identical to the sequence of 6 isolates responsible for the second wave of infections that occurred in Western and Central Kenya from October 2006 to January 2007, suggesting that ASFV virus exchange between the two countries might have occurred on more than one occasion (78). Therefore, movements of pigs, through trade, does play an important role in spreading ASFV beyond national borders.

Animal movement control in many countries is not fully regulated, and although there are policies in place, they lack enforcement. Informal trade of livestock is a concern in many countries, and there are several factors driving it (economical, social, political) (85). There are no physical markets for live pigs in most countries, especially in East Africa, a measure aimed at disease control. In Kenya and Uganda, buyers, mainly traders and middlemen, visit villages and farms in order to source pigs for further sale (29, 86). There are also opportunities for farmers to contact traders when they have a need to sell a pig, increasingly now relying on mobile phone technology. Pigs may be purchased daily and, when bought, are transported from the farm directly to the slaughter slab, either by herding or using other available means (bicycles, motorcycles, etc.) (73, 86, 87). Within East Africa, the marketing of pigs faces a number of challenges including non-compliance with regulations regarding movement control of animals and animal by-products, and poor transport infrastructure. This situation constitutes a high risk to the spread of ASF, given that in some cases suspected outbreaks of ASF are not revealed by farmers and traders in order to avoid losses, and to escape enforced restrictions by government authorities (19, 23). Other risky practices include the panic sale of sick pigs, the movement of traders and butchers from farm to farm without taking any biosecurity precautions, as they search for cheap animals, and the illegal transportation of animals between villages or districts without movement permits. In Uganda, pig traders were identified by value chain actors as the highest risk for ASF spread (26).

#### Pig Slaughtering and Processing

In SSA, pig slaughtering practices in most smallholder pig systems are generally poor, mainly due to lack of proper slaughter facilities. In some of the countries, there is usually no formal slaughtering place for pigs, and routine meat inspection by veterinary officers is rarely undertaken. In Uganda, for example, most of the time, pigs are slaughtered at the backyard and meat is either sold to the butchers or consumed at home to some extent (9). Several practices, related to informal slaughter of pigs, potentially contribute to spread of ASFV, including improper disposal of offals, often in the immediate environment (bush) and the use of slaughter waste for the feeding of live pigs and/or dogs (26, 88). The risk of ASFV being spread by butchers was compounded by use of poorly constructed slaughter slabs/sites with open drainage, ineffective or non-existent meat inspection services, lack of biosecurity measures, and sale of pork to customers who often are not aware of the risks of ASFV- infected pork (89).

### Factors Likely to Influence the Uptake of Biosecurity Measures

#### Lack of Knowledge and Lack of Awareness by Value Chain Actors

The implementation of biosecurity is key to successful pig production in an ASF-endemic environment (90). However, knowledge of the key principles of biosecurity is fundamental if farmers and other value chain actors such as traders, butchers and transporters want to substantially change their perception of disease risks, and consequently increase their level of awareness of the importance of biosecurity measures. In Uganda, several studies have recommended training of pig farmers on strict biosecurity measures as a means of mitigating ASF (16, 19). In Nigeria, the need for extension officers or livestock experts to educate less experienced farmers on pig production and provision of extension services aimed at raising technical knowledge on effective productivity and profitability was reported (91). Another study found that efficiency of pig production could be increased by 14% through farmer education and improving farming skills (92). A study in Chad highlighted the importance of providing knowledge to pig producers (93). In Uganda, participatory training can significantly improve farmer's knowledge of biosecurity (94). According to Young et al. (95), behavior change toward adopting improved biosecurity is likely to have positive benefits and impacts on the smallholder and public health at large. However, positively influencing the development of the smallholder farming system through uptake and adoption of sustainable interventions or change remains a major challenge, particularly with respect to improving the management of disease risks (96).

#### Financial Limitations of Smallholders in Sub-saharan Africa

In a specific smallholder pig sector such as Nigeria, according to Fasina et al. (90) additional workforce, costs and complexities of application of biosecurity, availability of funds are key barriers to adopt better practices. A study in Uganda concluded that pig farmers may be unwilling to adopt biosecurity practices if implemented alone to control ASF outbreaks unless there were financial incentives to compensate for higher costs (97). In Uganda, limited access of farmers to markets and the high cost of pig feed ingredients were among the major constraints of pigs farmers that interfere with the control of ASF (9). This situation may explain the reason why Costard et al. (98) advocate for market-based approaches or certification approaches to tackle ASF. However, according to Chenais et al. (61), causality of social and economic impact of ASF outbreaks in smallholder systems is complex. In the current pig systems context, farmers may rely on cheap biosecurity and animal management measures to sustain their pig enterprise; these practices are often not sufficient to prevent or control ASF. Profitability remains the principal driver for involvement in pig rearing, hence the understanding of this factor and its use in the introduction and maintenance of principles of biosecurity at farm level becomes important for controlling ASF in small- to medium-scale piggeries and farming communities (90). The assumption for promoting biosecurity is that compliance will lead to better performance and consequently higher financial returns; so that farmers can invest back in improving biosecurity in their farms and increase their pig production.

#### Socio-Cultural Factors

Knowledge levels, capacity, and incentives to adopt biosecurity measures at farm level are shaped by differences between men and women arising mainly from their socio-cultural backgrounds, responsibilities, and societal expectations. Pig farm tasks of men differ from those of women, depending on settings. Also, men and women have different knowledge, skills, experiences as well as needs and constraints (99). Decision making patterns in households are not homogenous, but cultural norms seem to influence certain patterns across most communities (100). In Uganda, women play a critical role in pig husbandry and biosecurity as they deal with most of the management activities (99, 101). Typical gender roles and the perceptions of men and women toward biosecurity undermine effective implementation of biosecurity measures in smallholder pig systems in Uganda. In most smallholder systems, given the crucial role women play in pig husbandry and disease control and the overall purpose of improving the livelihoods of smallholder pig keepers, interventions must address underlying gender inequalities and women's workload, which inhibit improved ASF control and prevention (102).

In Uganda, women reported facing constraints mostly related to labor demands that are time-consuming and also related to exposure to disease during the implementation of biosecurity. On the other hand, constraints faced by men are mostly periodic/occasional and related to social or community standing. On-farm constraints reported include lack of capital to construct pigsties and the purchase of farm tools/equipment, which is attributed to the low incomes of households, absence of alternative sources of revenue, and lack of labor to implement some biosecurity measures (e.g., for digging pits to bury dead pigs) (102). Addressing these issues would contribute toward creating an enabling environment for men and women to implement biosecurity measures. Engaging both female and male pig producers in ASF disease prevention and control can promote more sustainable livelihoods along the pig value chain and beyond. Through the provision of training for men and women relating to pig husbandry and disease control and through gender sensitization, gains can be made to increase the participation of men in taking on tasks that are, in the context of this setting, considered to be tasks of women (102). Training on biosecurity should explicitly target both men and women in households, reflect on the division of labor, open opportunities for women in emerging labor markets, and build on gender role changes that have already occurred rather than revert back to the traditional roles of women.

### ASF Control and Policy Implications

African swine fever was first detected in Kenya in 1910 but has since spread across the globe (30). Human behavior, livestock management, and inadequate biosecurity measures are the main factors driving its occurrence (103). In East Africa, the greatest risk is brought about by operations of traders, brokers and pig butchers (26). The challenge then is to identify practical, sustainable farmer-based and situation-specific solutions, and developing risk mitigation strategies along pig and pork- product value chains that will positively impact on the sector (30).

ASF is a transboundary animal disease (TAD) and building strong collaborations at national, regional and international levels is critical to its control. Disease spread to neighboring countries is mainly due to cross-border trade, either involving live pigs or pork products, formally and informally (104). The vast majority of TADs are highly contagious and usually have serious socio-economic impacts. Regional harmonization of ASF interventions is needed for the effective control of ASF (105). A global ASF strategy covering the period between 2020 and 2025 exists (103). This strategy considers the Global Framework for the Progressive Control of Transboundary Animal Diseases (GF-TADs) as a tool with the potential to fight transboundary animal diseases. GF-TADs is a joint initiative of FAO and OIE, with the expected participation of WHO for diseases with zoonotic potential, to achieve the prevention, detection and control of TADs (106). The Terrestrial animal health code requires importing countries to only accept animals that have been subjected to a health examination and which are accompanied by an international veterinary certificate (107).

A regional strategy for ASF control in SSA exists (105). It includes review and enforcement of existing disease control legislation and policies while promoting formulation, harmonization, and implementation where gaps are identified. The strategy proposes risk-based solutions that are feasible for outbreak control (105). Africa can learn a lot from approaches used to contain the disease in other countries. China issued several policies and regulations to prevent and control ASF outbreaks (64). This included establishing more stringent surveillance programs and the need to meet legal requirements relating to biosecurity. The Chinese Animal Husbandry and Veterinary Bureau formulated the animal vaccine regulation to counter the problem of fake veterinary vaccines on the market, that among other expectations requires those producing veterinary vaccines illegally to be fined 5 times the product value or RMB 200,000 in the case where the amount cannot be fixed (108). The EU adopted a directive that lays down community measures for the control of African swine fever. Measures required for reporting and follow up actions are very well-detailed. Member states were required to establish laws, regulations and administrative provisions necessary to comply with the relevant Directive (109).

Especially for Africa, institutional and legal elements that governments should consider when preparing for, and reacting to outbreaks of ASF, are already defined (110); some of which need to be in place before an outbreak occurs—for example existence of an emergency plan as an indication of preparedness, availability of funds, establishment of a legislative framework (assess current legislation and identify gaps), awareness creation (through the veterinary department, schools, the media etc.). Once an outbreak occurs, measures are put in place to check spread, legislation is enacted, and the public is sensitized. Being prepared for disease emergencies requires governments to set aside funds for this, including sampling of animals and laboratory confirmation of disease. Addressing the risk of ASF requires involvement and cooperation of all relevant stakeholders. This includes producers who are likely to comply with control measures if they are aware of the benefits that may result from such efforts (104). In a study by Dione et al. (94), veterinarians in Uganda were found not to always observe proper biosecurity measures when visiting pig farms. Inadequate enforcement of rules and regulations, obsolete legal frameworks, and lack of appropriate compensation schemes are the main regulatory challenges (105).

It is the responsibility of the veterinary authorities to control ASF, although stakeholder efforts are also required (111), including farmers who need to comply with biosecurity measures. But many SSA countries face a number of challenges including lack of political support and existence of policies that do not effectively respond to the needs of the sector (103, 105). With limited funds, control operations cannot be fully implemented. A starting point could be to lobby for increased government support, perhaps starting with sensitization at the lowest levels of governance. For ASF, mitigation strategies have to be effective and practical (112). Traders may be aware of how the disease clinically manifests (37) but factors including poor access to resources and policy limitations remain a problem (26). Improving biosecurity will require pig farmers to invest more resources into their pig businesses (105), a challenge given that these are low input/ low output operations. Confirmation of ASF is not a problem given that reference laboratories exist, however, for SSA, the main challenge has been the time it takes to detect the disease in the field (113). Early warning of disease relies on functional surveillance systems, rapid reporting and epidemiological analysis of results (49, 111). For countries where ASF is emerging, and to confine outbreaks, surveillance requires a more comprehensive policy, laboratory support and rapid response procedures and adequate human resources (63). Given the experience from recent outbreaks in China, investments in animal health system infrastructure, capacity building, and policy are needed to reduce the likelihood and costs associated with disease outbreaks (62).

In Kenya, reporting of notifiable diseases is well-defined in section 4 of the Animal Diseases Act (114). The Meat Control Act (114) requires animals to be inspected before slaughter. Pigs showing temperatures of 41°C or higher are supposed to be condemned. High fever is one of the symptoms of Africa swine fever. According to the pig Industry Act, Chapter 361 (repealed in 2006), pig farmers are required to have a license. It states that “*….every pig kept by a pig producer shall, whenever kept in a building, be confined in a pig-proof building and whenever not in a building shall be confined in a pig-proof paddock”*(115). In Uganda, construction of pig houses is specified in the regulations (116). Allowing pigs to roam freely is a concern, not only for ASF but also for diseases of public health importance (e.g., pig cysticercosis). The health status of the original herd will be lost when pigs mix with other pigs in animal markets (111) and will spread the virus to their new destination.

Many countries have regulations on animal movements, but enforcement of the measures has always been a challenge, especially in developing countries where food value chains are informal. In Kenya for example, moving animals from one county to another requires one to obtain a permit that the person accompanying the animals will need to carry and present to authorities when asked to do so. Appropriate incentives need to be determined to encourage compliance by relevant stakeholders. Although important for animal health and food safety, Livestock Identification and Traceability systems (LITS) are lacking in many developing countries. Namibia is an example of a country that has made progress in that regard (117). LITS is useful for disease management, vaccination programmes, husbandry, zoning or compartmentalization, surveillance, early response and notification systems, animal movement controls, inspection, certification, etc. (118), all of which are important in ASF control.

## Conclusions and Recommendations

Pigs are an important source of income especially to smallholder communities, and, with an increasing human population, can potentially mitigate risks of food insecurity. ASF remains the greatest threat to the pig sector, globally, and outbreaks can be devastating, especially in small farms who may not have other income sources. Although significant progress has been made in vaccine development, there has not been any breakthrough to date. Several control measures have been proposed and improved, but many of these are not designed in the context of developing countries. Although application of biosecurity measures can make a difference in these setting, compliance with even the simplest measures has been, and continues to be, a challenge, especially for farmers and other actors in the pig value chain. Relevant stakeholders need to be educated about the disease and implementation of biosecurity measures in an effort to mitigate the risks. However, farmers require options that are feasible and cheap to implement. Further research is needed to develop, validate, and sensitize farmers about these solutions, even as more research to develop vaccines continues. While stakeholder sensitization is an easy, short term investment, we recommend the development of a policy system that would ensure compliance with ASF control measures, while providing incentives to invest in the value chain. Interventions should be tailored to specific contexts and socio-economic environments if we want to boost adoption of biosecurity of smallholder pig value chain actors. In the context of COVID-19 epidemic, there is an opportunity to rethink the field of biosecurity taking into consideration a more integrated and holistic approach. This will encourage stakeholder engagement and also support smallholder producers.

## Author Contributions

All authors listed have made equal substantial, direct and intellectual contribution to the work, and approved it for publication.

## Conflict of Interest

The authors declare that the research was conducted in the absence of any commercial or financial relationships that could be construed as a potential conflict of interest.

## References

[1] Neumann C Harris DM Rogers LM. Contribution of animal source foods in improving diet quality and function in children in the developing world. Nutr Res. (2002) 22:193–220. 10.1016/S0271-5317(01)00374-8

[2] Thornton PK. Livestock production: recent trends, future prospects. Philos Trans R Soc B Biol Sci. (2010) 365:2853–67. 10.1098/rstb.2010.0134 20713389 PMC2935116

[3] Ampaire A Rothschild MF. Pigs, goats and chickens for rural development: small holder farmer's experience in Uganda. Livestock Res Rural Dev. (2010) 22:102. Available online at: http://www.lrrd.org/lrrd22/6/ampa22102.htm

[4] Phiri IK Ngowi H Afonso S Matenga E Boa M Mukaratirwa S. The emergence of Taenia solium cysticercosis in Eastern and Southern Africa as a serious agricultural problem and public health risk. Acta Trop. (2003) 87:13–23. 10.1016/s0001-706x(03)00051-2 12781374

[5] Delgado CL. Rising consumption of meat and milk in developing countries has created a new food revolution. J Nutr. (2003) 133(11 Suppl. 2):3907S–10S. 10.1093/jn/133.11.3907S 14672289

[6] FAO(2021). Sources of Meat. Animal Production and Health. Available online at: http://www.fao.org/ag/againfo/themes/en/meat/backgr_sources.html

[7] FAOSTAT. FAO Statistics Division. Food and Agriculture Organization of the United Nations (2010).

[8] FAO. Mapping Supply and Demand for Animal-Source Foods to 2030. Animal Production and Health Working Paper No. 2. Rome: FAO (2011).

[9] Ouma E Dione M Lule PM Pezo D Marshall K Roesel K. Smallholder pig value chain assessment in Uganda: results from producer focus group discussions and key informant interview. ILRI, Nairobi, Kenya (2015).

[10] Motsa'a JS Defang HF Keambou CT. Socio-economic and technical characteristics of pig (Sus scrofa domesticus) production system in the humid forest with monomodal rainfall agroecological zone of Cameroon. Int J Biol Chem Sci. (2018) 12 2318–27. 10.4314/ijbcs.v12i5.31

[11] Scheftelowitz M Thrän D. Unlocking the energy potential of manure—an assessment of the biogas production potential at the farm level in Germany. Agriculture. (2016) 6:20. 10.3390/agriculture6020020

[12] Deka R Thorpe WM Lapar L Kumar A. Assam's pig sub-sector: current status, constraints and opportunities. Assam: ILRI (2007).

[13] Dietze K. Pigs for Prosperity. Diversification Booklet Number 15. Rome: Rural Infrastructure and Agro-Industries Division Food and Agriculture Organization of the United Nations Rome (2011).

[14] Atuhaire DK Ochwo S Afayoa M Mwiine FN Kokas I Arinaitwe E. Epidemiological Overview of African Swine Fever in Uganda (2001–2012). J Vet Med. (2013). 2013:949638. 10.1155/2013/949638 26464916 PMC4590872

[15] Muhanguzi D Lutwama V Mwiine FN. Factors that influence pig production in Central Uganda—case study of Nangabo Sub-County, Wakiso district. Vet World. (2012) 5:346–51. 10.5455/vetworld.2012.346-351

[16] Dione MM Ouma EA Roesel K Kungu J Lule P Pezo D. Participatory assessment of animal health and husbandry practices in smallholder pig production systems in three high poverty districts in Uganda. Prev Vet Med. (2014) 117:565–76. 10.1016/j.prevetmed.2014.10.012 25458705

[17] Jolaoluwa Awosanya EJ Olugasa B Ogundipe G Grohn YT. Sero-prevalence and risk factors associated with African swine fever on pig farms in southwest Nigeria. BMC Vet Res. (2015) 11:133. 10.1186/s12917-015-0444-3 26063337 PMC4464725

[18] Foster JE. Chapter 8—Viruses as pathogens: animal viruses affecting wild and domesticated species. Viruses. Cambridge, MA: Academic Press. (2018). p. 189–216.

[19] Dione MM Akol J Roesel K Kungu J Ouma EA Wieland B. Risk factors for african swine fever in smallholder pig production systems in Uganda. Transbound Emerg Dis. (2015) 64:872–82. 10.1111/tbed.12452 26662861

[20] Nantima N Ocaido M Ouma E Davies J Dione M Okoth E. Risk factors associated with occurrence of African swine fever outbreaks in smallholder pig farms in four districts along the Uganda-Kenya border. Trop Anim Health Prod. (2015) 47:589–95. 10.1007/s11250-015-0768-9 25616986

[21] Thomas LF Bishop RP Onzere C McIntosh MT Lemire KA de Glanville WA. Evidence for the presence of African swine fever virus in an endemic region of Western Kenya in the absence of any reported outbreak. BMC Vet Res. (2016) 12:192. 10.1186/s12917-016-0830-5 27608711 PMC5016997

[22] Okoth EA Onzere C Amimo JO Riitho V Mwangi W Davies J. Detection of African swine fever virus in the tissues of asymptomatic pigs in smallholder farming systems along the Kenya–Uganda border: implications for transmission in endemic areas and ASF surveillance in East Africa. J Gen Virol. (2017) 98:1806–14. 10.1099/jgv.0.000848 28721858

[23] Nantima N Davies J Dione M Ocaido M Okoth E Mugisha A. Enhancing knowledge and awareness of biosecurity practices for control of African swine fever among smallholder pig farmers in four districts along the Kenya-Uganda border. Trop Anim Health Prod. (2016) 48:727–34. 10.1007/s11250-016-1015-8 26922740

[24] Dione M Nantima N Mayega L Amia W Wieland B Ouma E. Enhancing biosecurity along Uganda's pig value chains to control and prevent African swine fever. Livestock Brief 1. Nairobi: ILRI (2017).

[25] Barongo MB Bishop RP Fèvre EM Knobel DL Ssematimba A. A mathematical model that simulates control options for african swine fever virus (ASFV). PLoS ONE. (2016) 11:e0158658. 10.1371/journal.pone.0158658 27391689 PMC4938631

[26] Dione M Ouma E Opio F Kawuma B Pezo D. Qualitative analysis of the risks and practices associated with the spread of African swine fever within the smallholder pig value chains in Uganda. Prev Vet Med. (2016) 135:102–12. 10.1016/j.prevetmed.2016.11.001 27931922

[27] Penrith ML. African swine fever. Onderstepoort J Vet Res. (2009) 76:91–5. 10.4102/ojvr.v76i1.70 19967933

[28] Revilla Y Pérez-Núñez D Richt JA. African swine fever virus biology and vaccine approaches. Adv Virus Res. (2018) 100:41–74. 10.1016/bs.aivir.2017.10.002 29551143

[29] Mutua FK Dewey CE Arimi SM Ogara WO Githigia SM Levy M. Indigenous pig management practices in rural villages of Western Kenya. Livestock Res Rural Dev. (2011) 23:144. Available online at: http://www.lrrd.org/lrrd23/7/mutu23144.htm

[30] Penrith ML. Current status of African swine fever. CABI Agric Biosci. (2020) 1:11. 10.1186/s43170-020-00011-w31538406

[31] Wabacha JK Maribei JM Mulei CM Kyule MN Zessin KH Oluoch-Kosura W. Characterisation of smallholder pig production in Kikuyu Division, central Kenya. Prev Vet Med. (2004) 63:183–95. 10.1016/j.prevetmed.2004.02.007 15158570

[32] Karimuribo ED Chenyambuga SW Makene VW Mathias S. Livestock research for rural development. Characteristics and production constraints of rural-based small-scale pig farming in Iringa region, Tanzania. Livestock Res Rural Dev. (2011) 23:2011*23.

[33] Kimbi E Lekule F Mlangwa J Mejer H Thamsborg S. Smallholder pigs production systems in Tanzania. J Agric Sci Technol A. (2015) 5:47–60. 10.17265/2161-6256/2015.01A.007

[34] Nantima N Ocaido M Davies J Dione M Okoth E Mugisha A. Characterization of smallholder pig production systems in four districts along the Uganda-Kenya border. Livestock Res Rural Dev. (2015) 27:166.

[35] Mbuza F Majyambere D Ayabagabao JDD Dutuze AF. Inventory of pig production systems in Rwanda. Int J Livestock Prod. (2016) 7:41–7. 10.5897/IJLP2016.0299

[36] Dione M Masembe C Akol J Amia W Kungu J Lee HS. The importance of on-farm biosecurity: sero-prevalence and risk factors of bacterial and viral pathogens in smallholder pig systems in Uganda. Acta Trop. (2018) 187:214–21. 10.1016/j.actatropica.2018.06.025 29949731

[37] Atherstone C Galiwango RG Grace D Alonso S Dhand NK Ward MP. Analysis of pig trading networks and practices in Uganda. Trop Anim Health Prod. (2019) 51:137–47. 10.1007/s11250-018-1668-6 30073452 PMC6347582

[38] Galindo I Alonso C. African swine fever virus: a review. Viruses. (2017) 9:103. 10.3390/v9050103 PMC5454416 28489063

[39] Dixon LK Sun H Roberts H. African swine fever. Antiviral Res. (2019) 165:34–41. 10.1016/j.antiviral.2019.02.018 30836106

[40] Jori F Vial L Penrith ML Pérez-Sánchez R Etter E Albina E. Review of the sylvatic cycle of African swine fever in sub-Saharan Africa and the Indian ocean. Virus Res. (2013) 173:212–27. 10.1016/j.virusres.2012.10.005 23142551

[41] Murcia P Donachie W Palmarini M. Viral pathogens of domestic animals and their impact on biology, medicine and agriculture. Encyclopedia of Microbiol. (2009). p. 805–19.

[42] Sánchez-Vizcaíno JM Mur L. African swine fever diagnosis update. Dev Biol. (2013) 135:159–65. 10.1159/000189240 23689893

[43] Bastos ADS Fasina FO King DP. African swine fever (Chapter 50). In: Liu D, editor. Manual of Security Sensitive Microbes and Toxins. Taylor and Francis CRC Press (2014). p. 579–87.

[44] Bastos ADS Fasina FO King DP. African swine fever (Chapter 50). In: Liu D, editor. Manual of Security Sensitive Microbes and Toxins. Taylor and Francis; *CRC Press*, (2014). p. 579–87.

[45] Rock DL. Challenges for African swine fever vaccine development— “… perhaps the end of the beginning”.. Vet Microbiol. (2017) 206:52–8. 10.1016/j.vetmic.2016.10.003 27756505

[46] Jurado C Martínez-Avilés M De La Torre A Štukelj M de Carvalho Ferreira HC Cerioli M. Relevant measures to prevent the spread of african swine fever in the european union domestic pig sector. Front Vet Sci. (2018) 5:77. 10.3389/fvets.2018.00077 29713637 PMC5912175

[47] Mutua F Lindahl J Randolph D. Possibilities of establishing a smallholder pig identification and traceability system in Kenya. Trop Anim Health Prod. (2020) 52:859–70. 10.1007/s11250-019-02077-9 31529303 PMC7039844

[48] Beltrán-Alcrudo D Arias M Gallardo C Kramer S Penrith ML. African swine fever: detection and diagnosis—a manual for veterinarians. FAO Animal Production and Health Manual No. 19. Rome: Food and Agriculture Organization of the United Nations (FAO) (2017). p. 88.

[49] FAO. Preparation of African swine fever contingency plans. Penrith ML Guberti V Depner K Lubroth J editor. FAO Animal Production and Health Manual No. 8. Rome: FAO (2009).

[50] Nespeca R Vaillancourt JP Morrow WE. Validation of a poultry biosecurity survey. Prev Vet Med. (1997) 31:73–86. 10.1016/S0167-5877(96)01122-1 9234427

[51] Carr J Howells M. Biosecurity on pig and poultry farms: principles for the veterinary profession. In Pract. (2018) 40:238–48. 10.1136/inp.k2593

[52] Dargatz DA Garry FB Traub-Dargatz JL. An introduction to biosecurity of cattle operations. Vet Clin North Am Food Anim Pract. (2002) 18:1–5. 10.1016/S0749-0720(02)00002-6 12064163

[53] Villarroel A Dargatz DA Lane VM McCluskey BJ Salman MD. Suggested outline of potential critical control points for biosecurity and biocontainment on large dairy farms. J Am Vet Med Assoc. (2007) 6:808–19. 10.2460/javma.230.6.808 17362152

[54] Laanen M Persoons D Ribbens S De Jong E Callens B Strubbe M. Relationship between biosecurity and production/antimicrobial treatment characteristics in pig herds. Vet J. (2013) 198:508–12. 10.1016/j.tvjl.2013.08.029 24268483

[55] FAO OIE WB. Good practices for biosecurity in the pig sector—issues and options in developing and transition countries. FAO Animal Production and Health Paper No. 169. Rome: FAO (2010).

[56] Ford WB. Disinfection procedures for personnel and vehicles entering and leaving contaminated premises. Rev Sci Tech Off Int Epizoot. (1995) 14:393–401. 10.20506/rst.14.2.847 7579638

[57] De Lorenzi G Borella L Alborali GL Prodanov-Radulović J Štukelj M Bellini S. African swine fever: a review of cleaning and disinfection procedures in commercial pig holdings. Res Vet Sci. (2020) 132:262–7. 10.1016/j.rvsc.2020.06.009 32693250

[58] PREP F. NAHEMS Guidelines: Cleaning and Disinfection. Ames, IA, MD: The Foreign Animal Disease Preparedness and Response Plan/National Animal Health Emergency Management System. (2014).

[59] Chenais E Sternberg-Lewerin S Boqvist S Liu L LeBlanc N Aliro T. African swine fever outbreak on a medium-sized farm in Uganda: biosecurity breaches and within-farm virus contamination. Trop Anim Health Prod. (2017) 49:337–46. 10.1007/s11250-016-1197-0 27966070 PMC5253150

[60] Mulumba-Mfumu LK Saegerman C Dixon LK Madimba KC Kazadi E Mukalakata NT. African swine fever: Update on Eastern, Central and Southern Africa. Transbound Emerg Dis. (2019) 66:1462–80. 10.1111/tbed.13187 30920725

[61] Chenais E Boqvist S Emanuelson U von Bromssen C Ouma E Aliro T. Quantitative assessment of social and economic impact of African swine fever outbreaks in northern Uganda. Prev Vet Med. (2017) 144:134–48. 10.1016/j.prevetmed.2017.06.002 28716195

[62] Weaver TRD Habib N. Evaluating Losses Associated with African Swine Fever in the People's Republic of China and Neighboring Countries. ADB East Asia Working Paper Series No. (2020) 27:38. 10.22617/WPS200263-2

[63] Woonwong Y Do Tien D Thanawongnuwech R. The future of the pig industry after the introduction of african swine fever into Asia. Anim Front. (2020) 10:30–7. 10.1093/af/vfaa037 33150009 PMC7596796

[64] Tao D Sun D Liu Y Wei S Yang Z An T. One year of African swine fever outbreak in China. Acta Trop. (2020) 211:105602. 10.1016/j.actatropica.2020.105602 32598922

[65] Berthe F. The Global Economic Impact of ASF. Paris: OIE; Bulletin Panorama (2020).

[66] Mason-D'Croz D Bogard JR Herrero M Robinson S Sulser TB Wiebe K. Modelling the global economic consequences of a major African swine fever outbreak in China. Nat Food. (2020) 1:221–8. 10.1038/s43016-020-0057-2 33634268 PMC7116817

[67] Yona CM Vanhee M Simulundu E Makange M Nauwynck HJ Misinzo G. Persistent domestic circulation of African swine fever virus in Tanzania, 2015–2017. BMC Vet Res. (2020) 16:369. 10.1186/s12917-020-02588-w 33004025 PMC7528248

[68] Swai ES Lyimo CJ. Impact of African Swine fever epidemics in smallholder pig production units in Rombo district of Kilimanjaro, Tanzania. Livestock Res Rural Dev. (2014) 26:32. Available online at: http://www.lrrd.org/lrrd26/2/SWAI26032.html

[69] Niemi JK. Impacts of African swine fever on pigmeat markets in Europe. Front Vet Sci. (2020) 7:634. 10.3389/fvets.2020.00634 33062656 PMC7518409

[70] Costard S Zagmutt FJ Porphyre T Pfeiffer DU. Small-scale pig farmers' behavior, silent release of African swine fever virus and consequences for disease spread. Sci Rep. (2015) 5:17074. 10.1038/srep17074 26610850 PMC4661460

[71] Roesel K Dohoo I Baumann M Dione M Grace D Clausen PH. Prevalence and risk factors for gastrointestinal parasites in small-scale pig enterprises in Central and Eastern Uganda. Parasitol Res. (2017) 116:335–45. 10.1007/s00436-016-5296-7 27785599 PMC5167772

[72] Rutebarika C Ademun AR. African swine fever Diagnostics, surveillance, epidemiology and control in Uganda. In: Identification of Researchable Issues Targeted to the Endemic Areas Within Sub-Saharan Africa. Nairobi: Fairview Hotel (2011).

[73] Ouma EA Dione MM Lule PM Roesel K Pezo D. Characterization of smallholder pig production systems in Uganda: constraints and opportunities for engaging with market systems. Livestock Res Rural Dev. (2014) 26:56. Available online at: http://www.lrrd.org/lrrd26/3/ouma26056.htm

[74] Adjei OD Osei-Amponsah R Ahunu BK. Characterization of local pig production systems in Ghana. Bull Anim Health Prod Africa. (2015) 63:337–42.

[75] Mutua FK Dewey C Arimi S Ogara W Levy M Schelling E. A description of local pig feeding systems in village smallholder farms of Western Kenya. Trop Anim Health Prod. (2012) 44:1157–62. 10.1007/s11250-011-0052-6 22219174

[76] Bellini S Casadei G De Lorenzi G Tamba M. A review of risk factors of african swine fever incursion in pig farming within the european union scenario. Pathogens. (2021) 10:84. 10.3390/pathogens10010084 33478169 PMC7835761

[77] Perith ML Guberti G Lubroth J. Preparation of African Swine fever contingency plan. Rome: FAO (2009).

[78] Gallardo C Ademun AR Nieto N Nantima N Arias M Martín E. Genotyping of African swine fever virus (ASFV) isolates associated with disease outbreaks in Uganda in 2007. Afr J Biotechnol. (2011) 10:3488–97. 10.5897/AJB10.1439

[79] Niederwerder MC Stoian AMM Rowland RRR Dritz SS Petrovan V Constance LA. Infectious dose of African swine fever virus when consumed naturally in liquid or feed. Emerg Infect Dis. (2019) 25:891–7. 10.3201/eid2505.181495 30761988 PMC6478231

[80] Olševskis E Guberti V SerŽants M Westergaard J Gallardo C Rodze I. African swine fever virus introduction into the EU in 2014: Experience of Latvia. Res Vet Sci. (2016) 105 28–30. 10.1016/j.rvsc.2016.01.006 27033903

[81] Dee SA Bauermann FV Niederwerder MC Singrey A Clement T de Lima M. Survival of viral pathogens in animal feed ingredients under transboundary shipping models. PLoS ONE. (2018) 13:e0194509. 10.1371/journal.pone.0194509 29558524 PMC5860775

[82] Costard S Mur L Lubroth J Sanchez-Vizcaino JM Pfeiffer DU. Epidemiology of African swine fever virus. Virus Res. (2012)173:1–7. 10.1016/j.virusres.2012.10.030 23123296

[83] Tejler E. Outbreaks of African swine fever in domestic pigs in Gulu district, Uganda (Examensarbete). Swedish University of Agricultural Sciences, Examensarbete inom veterinärprogrammet, Uppsala. (2012).

[84] Okoth E Gallardo C Macharia JM Omore A Pelayo V Bulimo DW. Comparison of African swine fever virus prevalence and risk in two contrasting pig-farming systems in South-west and Central Kenya. Prev Vet Med. (2013) 110:198–205. 10.1016/j.prevetmed.2012.11.012 23219357

[85] Grace D Little P. Informal trade in livestock and livestock products. Rev Sci Tech Off Int Epiz. (2020) 39:183–92. 10.20506/rst.39.1.3071 32729569

[86] Mtimet N Baker D Ouma E. Analysing pig traders in Uganda: sampling issues, marketing activities, and constraint analysis. Dev Pract. (2018) 28:107–24. 10.1080/09614524.2017.1363873

[87] Levy M Dewey C Weersink A Mutua F Poljak Z. Pig marketing and factors associated with prices and margins in Western Kenya. J Agric Econ Dev. (2013) 2:371–83.

[88] Muhangi D Masembe C Berg M Ståhl K Ocaido M. Practices in the pig value chain in Uganda; implications to African swine fever transmission. Livestock Res Rural Dev. (2014) 26:94. Available online at: https://www.lrrd.cipav.org.co/lrrd26/5/muha26094.htm

[89] Lichoti JK Davies J Maru Y Kitala PM Githigia SM Okoth E. Pig traders' networks on the Kenya-Uganda border highlight potential for mitigation of African swine fever virus transmission and improved ASF disease risk management. Prev Vet Med. (2017) 140:87–96. 10.1016/j.prevetmed.2017.03.005 28460754

[90] Fasina FO Lazarus DD Spencer BT Makinde AA Bastos AD. Cost implications of African swine fever in smallholder farrow-to-finish units: economic benefits of disease prevention through biosecurity. Transbound Emerg Dis. (2012) 59:244–55. 10.1111/j.1865-1682.2011.01261.x 21929615

[91] Umeh JC Ogbanje C Adejo MA. Technical efficiency analysis of pig production: a sustainable animal protein augmentation for Nigerians. J Adv Agric Technol. (2015) 2:19–24. 10.12720/joaat.2.1.19-24

[92] Obayelu AE Ogunmola OO Sowande OK. Economic analysis and the determinants of pig production in ogun state, Nigeria. Agricultura Tropica et Tubtropica. (2017) 50/2:61–70. 10.1515/ats-2017-0007

[93] Youssouf ML Zeuh V Adoum IY Kabore CY. Production practices and constraints in pig farming in N'Djamena area, Tchad. Int J Livestock Prod. (2014) 5:196–203.

[94] Dione M Dohoo I Ndiwa N Poole J Ouma E Amia W. Impact of participatory training on farmers' knowledge, attitude and practices of biosecurity measures for the control of African swine fever in Uganda. Transbound Emerg Dis. (2020) 67:1–12. 10.1111/tbed.13587 32311216 PMC7754142

[95] Young JR Suon S Andrews CJ Henry LA Windsor PA. Assessment of financial impact of FMD on smallholder cattle farmers in southern Cambodia. Transbound Emerg Dis. (2013) 60:166–74. 10.1111/j.1865-1682.2012.01330.x 22510453

[96] Young JR Evans-Kocinski S Bush RD Windsor PA. Improving smallholder farmer biosecurity in the mekong region through change management. Transbound Emerg Dis. (2015) 62:491–504. 10.1111/tbed.12181 26302253

[97] Ouma E Dione M Birungi R Lule P Lawrence M Dizyee K. African swine fever control and market integration in Ugandan peri-urban smallholder pig value chains: An ex-ante impact assessment of interventions and their interaction. Prev Vet Med. (2018) 151:29–39. 10.1016/j.prevetmed.2017.12.010 29496103

[98] Costard S Zagmutt F Porphyre T Roger F Pfeiffer DU. Small-scale pig farmers, behaviour, silent release of African swine fever and consequences for persistence. In: International Symposia on Veterinary, Epidemiology, and Economics 2012; Maastricht, Netherlands: ISVEE; (2012).

[99] Ouma E Ochago R Dione M Birungi R Lule P. Gender equitable pig business hubs. In: Rhiannon P Eerdewijk A, editors. A Different Kettle of Fish? Gender Integration in Livestock and Fish Research. Volendam: LM Publishers (2016).

[100] FAO. FAO corporate document repository. Meat processing technology for small- to medium-scale producers. Rome. (2013). Avaialble online at: http://www.fao.org/3/ai407e/ai407e.pdf (accessed December 30, 2013).

[101] Dione M Ochago R Ouma E Lule P Birungi B. The gender dimensions of a pig disease: African swine fever in Uganda. In: Rhiannon P Eerdewijk A, editors. A Different Kettle of Fish? Gender Integration in Livestock and Fish Research. Volendam: LM Publishers (2016).

[102] Dione M Ochago R Ouma E Lule P Kakinda MJ Nyapendi R. Gender perspective of pig husbandry and disease control among smallholder pig farmers in Uganda. AgriGender. (2020) 5:13–26. 10.19268/JGAFS.522020.2

[103] FAO OIE. Global control of African swine fever: A GF-TADs initiative. 2020–2025. Paris (2020). Available online at: https://www.oie.int/fileadmin/Home/eng/Animal_Health_in_the_World/docs/pdf/ASF/ASF_GlobalInitiative_web.pdf 10.20506/bull.2020.1.3116 (accessed March, 2021).

[104] Costard S Wieland B de Glanville W Jori F Rowlands R Vosloo W. African swine fever: how can global spread be prevented? Philos Trans R Soc Lond B Biol Sci. (2009) 364:2683–96. 10.1098/rstb.2009.0098 19687038 PMC2865084

[105] FAO AU-IBAR ILRI. Regional strategy for the control of african swine fever in Africa (2017). Available online at: https://rr-africa.oie.int/wp-content/uploads/2020/01/au_strategy_asf_en.pdf (accessed March, 2021).

[106] FAO. Global Framework for the Progressive Control of Transboundary Animal Diseases (GF-TADs). Rome: FAO (2021). Available online at: http://www.gf-tads.org/about/en/

[107] OIE. Terrestrial Animal Health Code. vol I. 20th ed. Paris: OIE (2011).

[108] Ding Y Wang Y. Big government: The fight against the African Swine Fever in China. J Biosafe Biosecur. (2020) 2:44–9. 10.1016/j.jobb.2020.04.001

[109] EU. Council Directive 2002/60/EC of 27 June 2002 laying down specific provisions for the control of African swine fever and amending Directive 92/119/EEC as regards Teschen disease and African swine fever (Text with EEA relevance) (2002). Available online at: https://eur-lex.europa.eu/eli/dir/2002/60/oj (accessed March, 2021).

[110] FAO Institutional Legal Measures To Combat African Swine Fever. (1999). Available online at: http://www.fao.org/3/bb036e/bb036e.pdf (accessed March, 2021).

[111] Bellini S Rutili D Guberti V. Preventive measures aimed at minimizing the risk of African swine fever virus spread in pig farming systems. Acta Vet Scand. (2016) 58:82–82. 10.1186/s13028-016-0264-x 27899125 PMC5129245

[112] Guinat C Gogin A Blome S Keil G Pollin R Pfeiffer DU. Transmission routes of African swine fever virus to domestic pigs: current knowledge and future research directions. Vet Rec. (2016) 178:262–7. 10.1136/vr.103593 26966305 PMC4819659

[113] Sánchez-Vizcaíno JM. Early detection and contingency plans for African swine fever. In: Conf. Paris: OIE. (2010).

[114] GOK. Meat Control (Local Slaughterhouse) Regulations, 2010 Arrangement of Regulations (2012). Available online at: http://extwprlegs1.fao.org/docs/pdf/ken101239.pdf (accessed March, 2021).

[115] GOK. Animal Diseases Act Chapter 364. (1989). National Council for Law Reporting with the Authority of the Attorney-General (2012). Available online at: http://extwprlegs1.fao.org/docs/pdf/ken63506.pdf

[116] Go U. The Animal Diseases Act (2015). Statutory Instrument 38−4. The Animal Diseases Rules. Arrangement of Rules. Available online at: https://ugandatrades.go.ug/media/136_1Animal_Diseases_Rules_(S.I._38_4).pdf (accessed March, 2021).

[117] Prinsloo T de Villiers C. A framework to define the impact of sustainable ICT for agriculture projects: the Namibian livestock traceability system. EJISDC. (2017) 86:1–22. 10.1002/j.1681-4835.2017.tb00606.x

[118] OIE. General principles on identification and traceability of live animals Terrestrial Animal Health Code - 28/06/2019 (2019). Available online at: https://www.oie.int/fileadmin/home/eng/health_standards/tahc/current/chapitre_ident_traceability.pdf (accessed March, 2021).

